# Supplementary figures and images for: ATP-release pannexin channels are gated by lysophospholipids
Source: eLife. 2025 May 1;14:RP107067. doi: 10.7554/eLife.107067 (PMC12045621; doi:10.7554/eLife.107067)

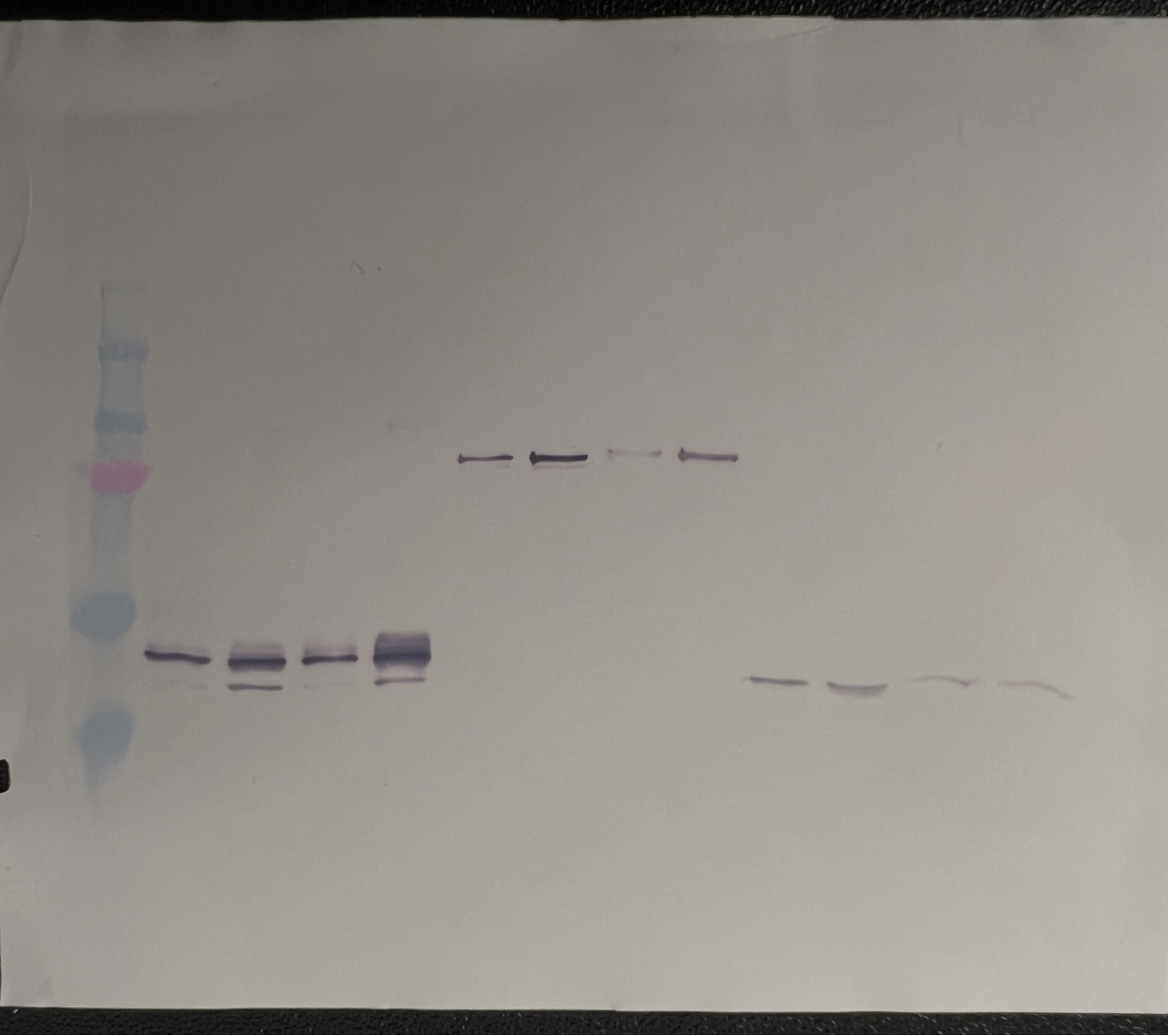

Supplement: Figure 1—figure supplement 1—source data 2. [file elife-107067-fig1-figsupp1-data2.zip › Fig1_Source_data4_raw/Fig1_SD4-raw1.png]

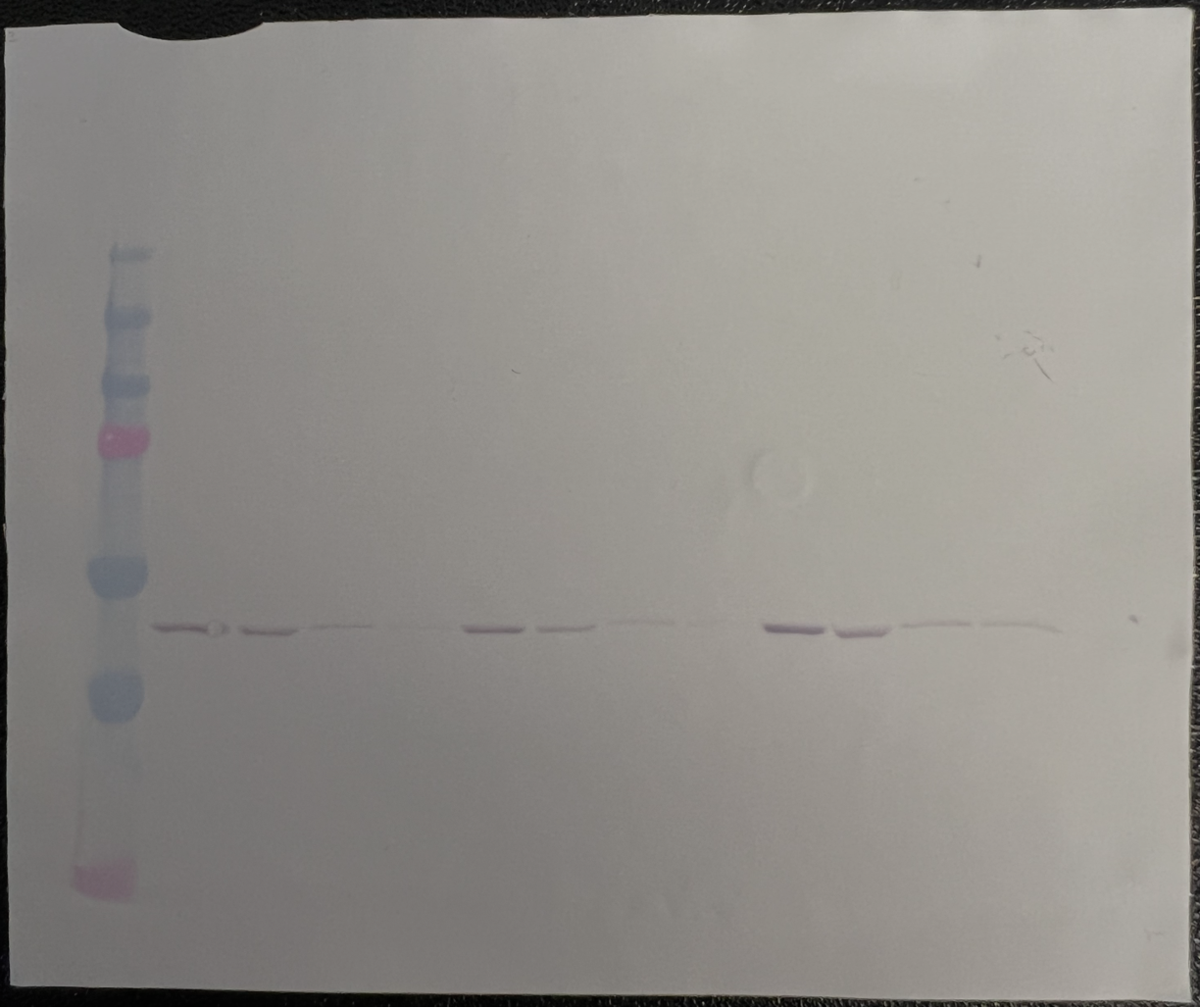

Supplement: Figure 1—figure supplement 1—source data 2. [file elife-107067-fig1-figsupp1-data2.zip › Fig1_Source_data4_raw/Fig1_SD4-raw2.png]

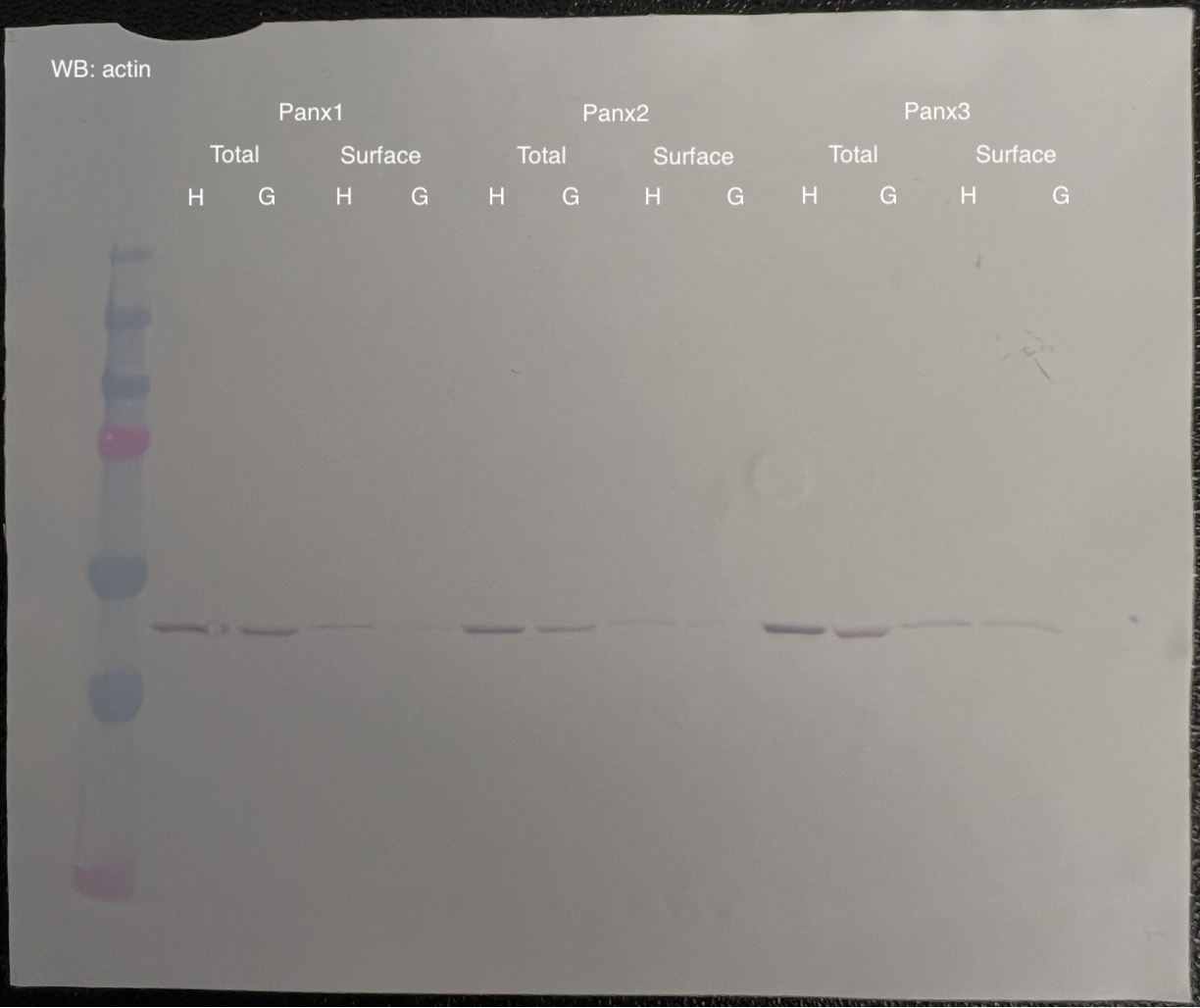

Supplement: Figure 1—figure supplement 1—source data 3. [file elife-107067-fig1-figsupp1-data3.zip › Fig1_Source_data5_annotated/Fig1_SD5-annotated2.png]

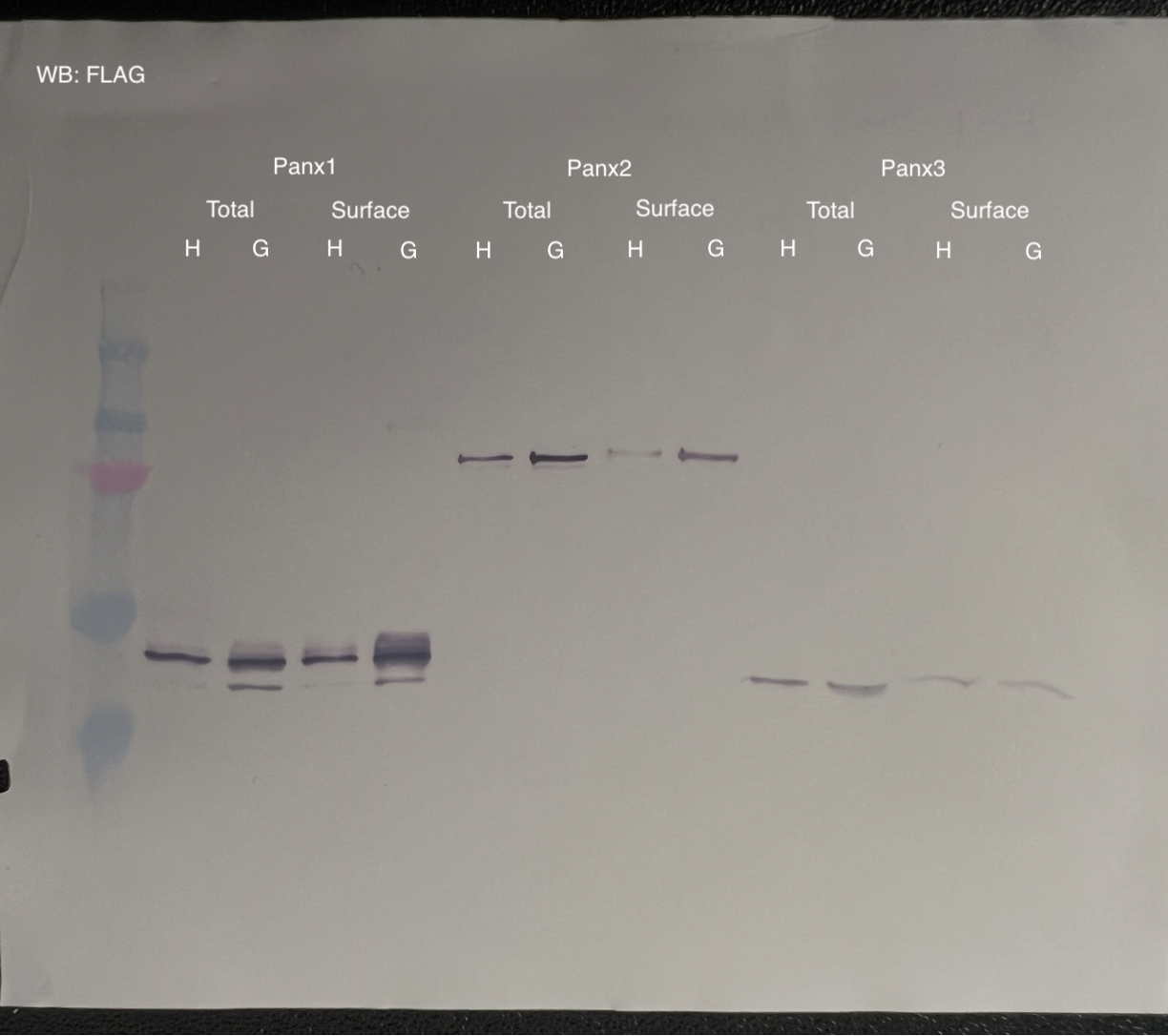

Supplement: Figure 1—figure supplement 1—source data 3. [file elife-107067-fig1-figsupp1-data3.zip › Fig1_Source_data5_annotated/Fig1_SD5-annotated1.png]

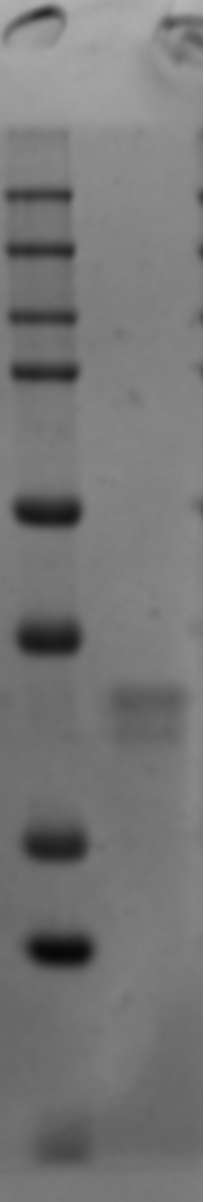

Supplement: Figure 4—figure supplement 1—source data 2. [file elife-107067-fig4-figsupp1-data2.zip › Fig4_Source_data3_raw/Fig4_SD3-raw1.png]

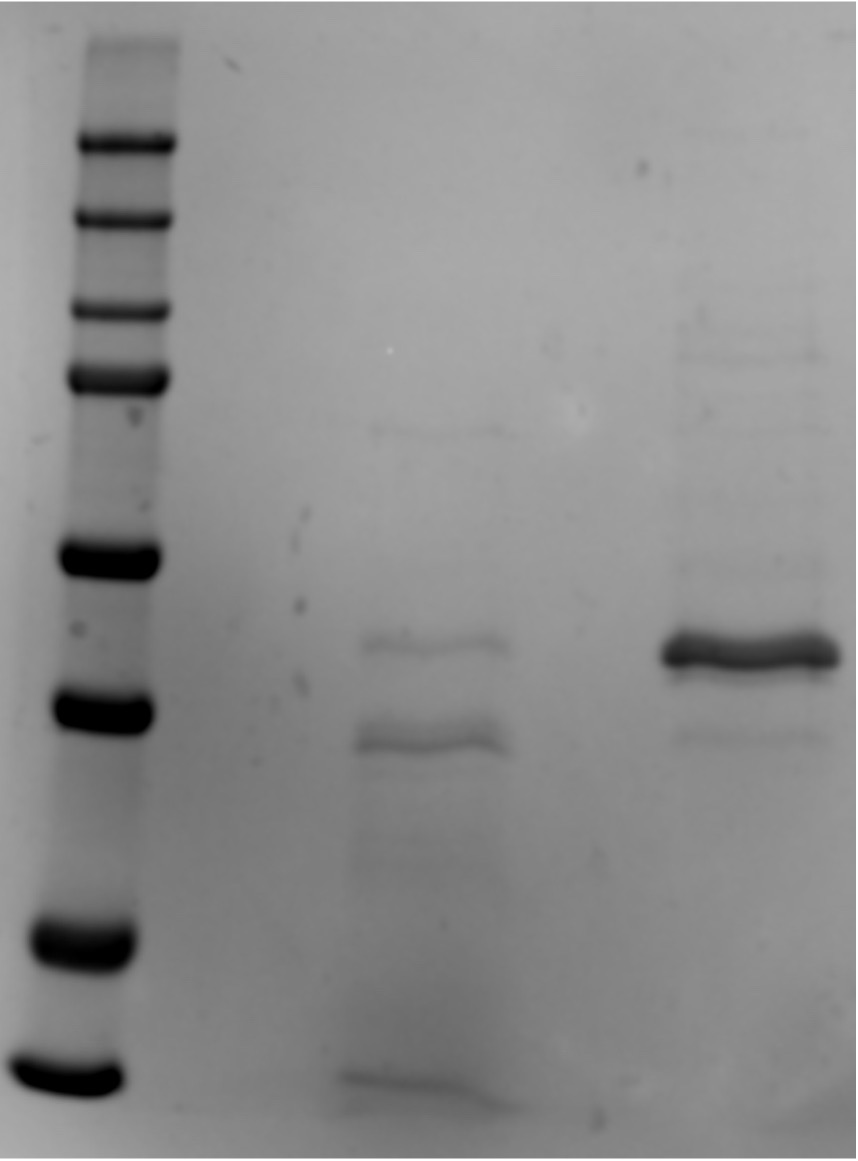

Supplement: Figure 4—figure supplement 1—source data 2. [file elife-107067-fig4-figsupp1-data2.zip › Fig4_Source_data3_raw/Fig4_SD3-raw1.jpg]

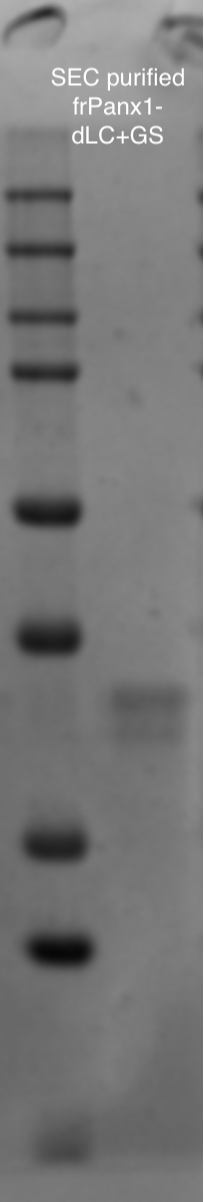

Supplement: Figure 4—figure supplement 1—source data 3. [file elife-107067-fig4-figsupp1-data3.zip › Fig4_Source_data4_annotated/Fig4_SD4-annotated1.png]

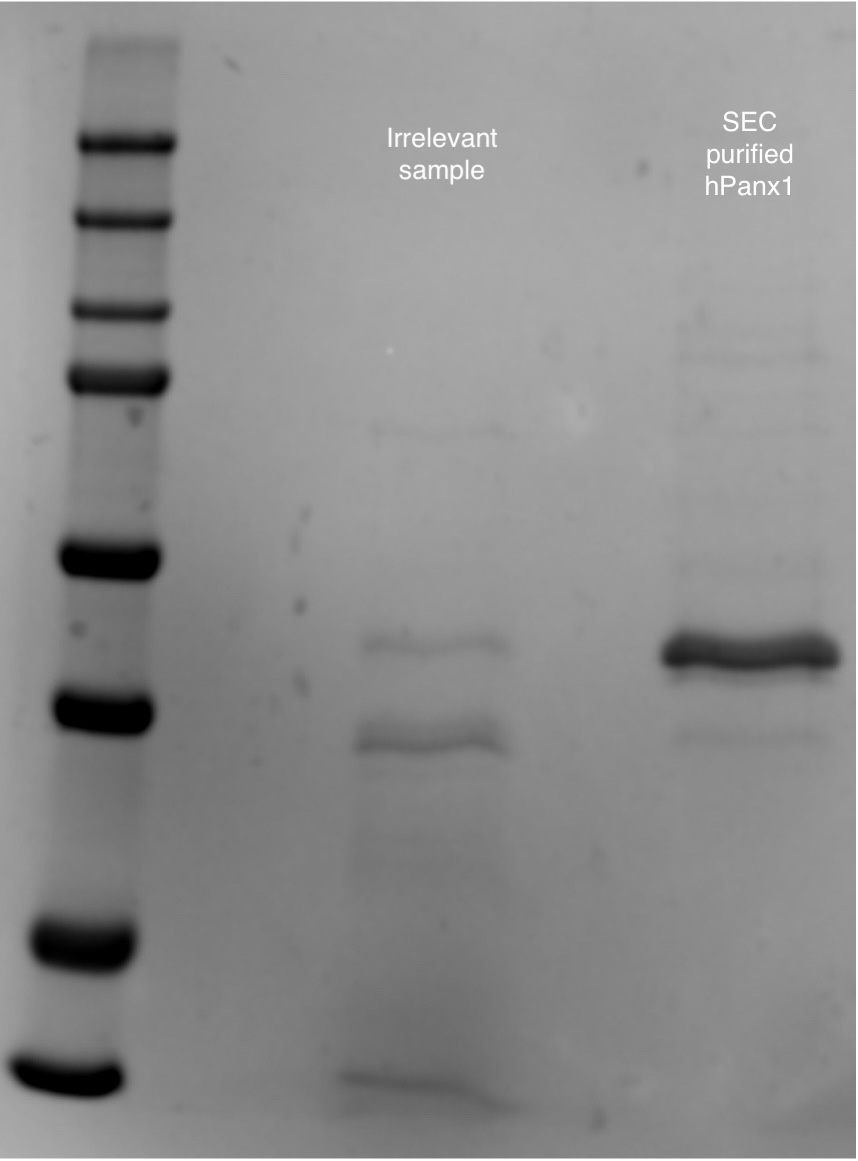

Supplement: Figure 4—figure supplement 1—source data 3. [file elife-107067-fig4-figsupp1-data3.zip › Fig4_Source_data4_annotated/Fig4_SD4-annotated1.jpg]

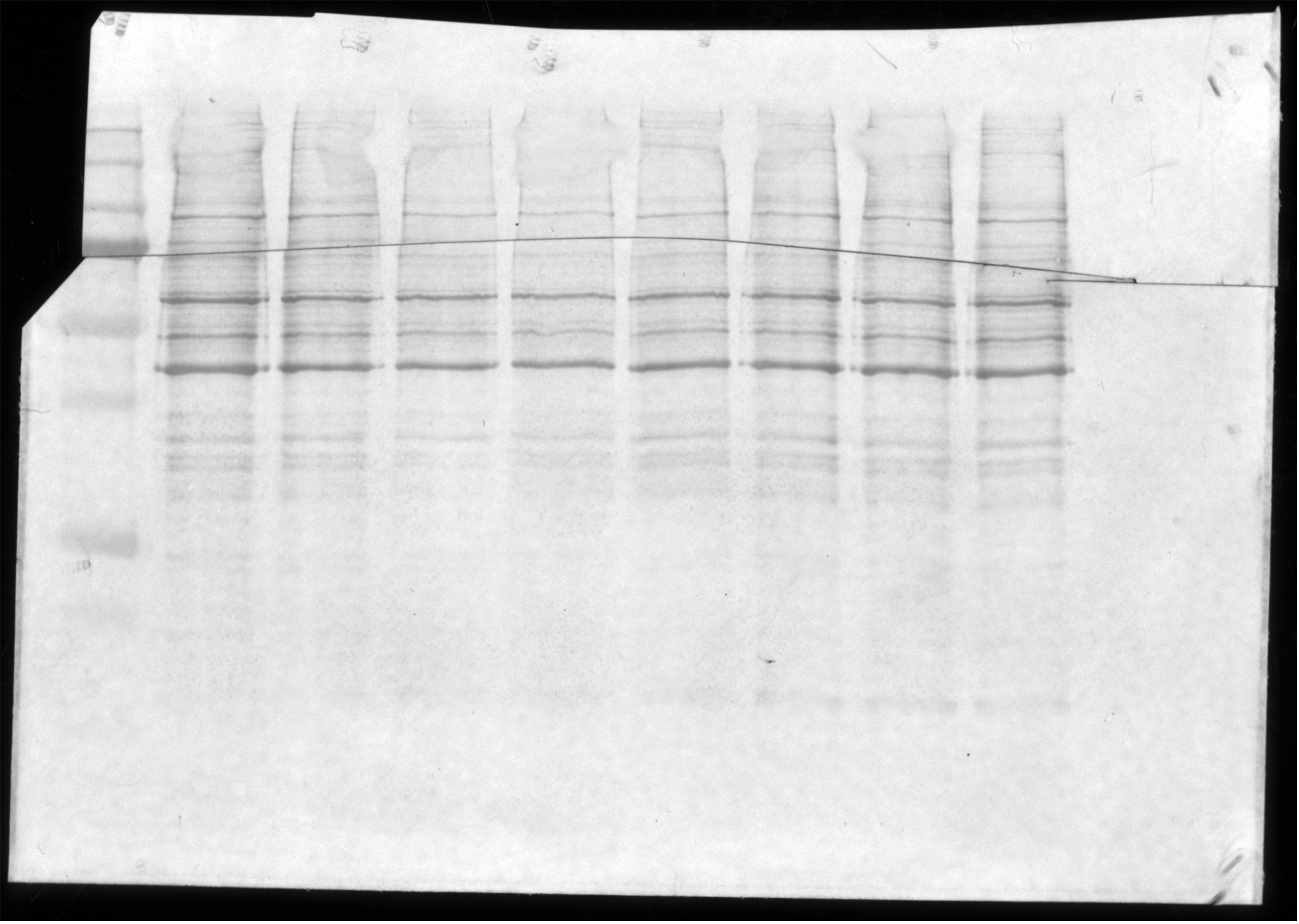

Supplement: Figure 6—source data 2. [file elife-107067-fig6-data2.zip › Fig6_Source_data2_raw/Fig6_SD2_raw5.png]

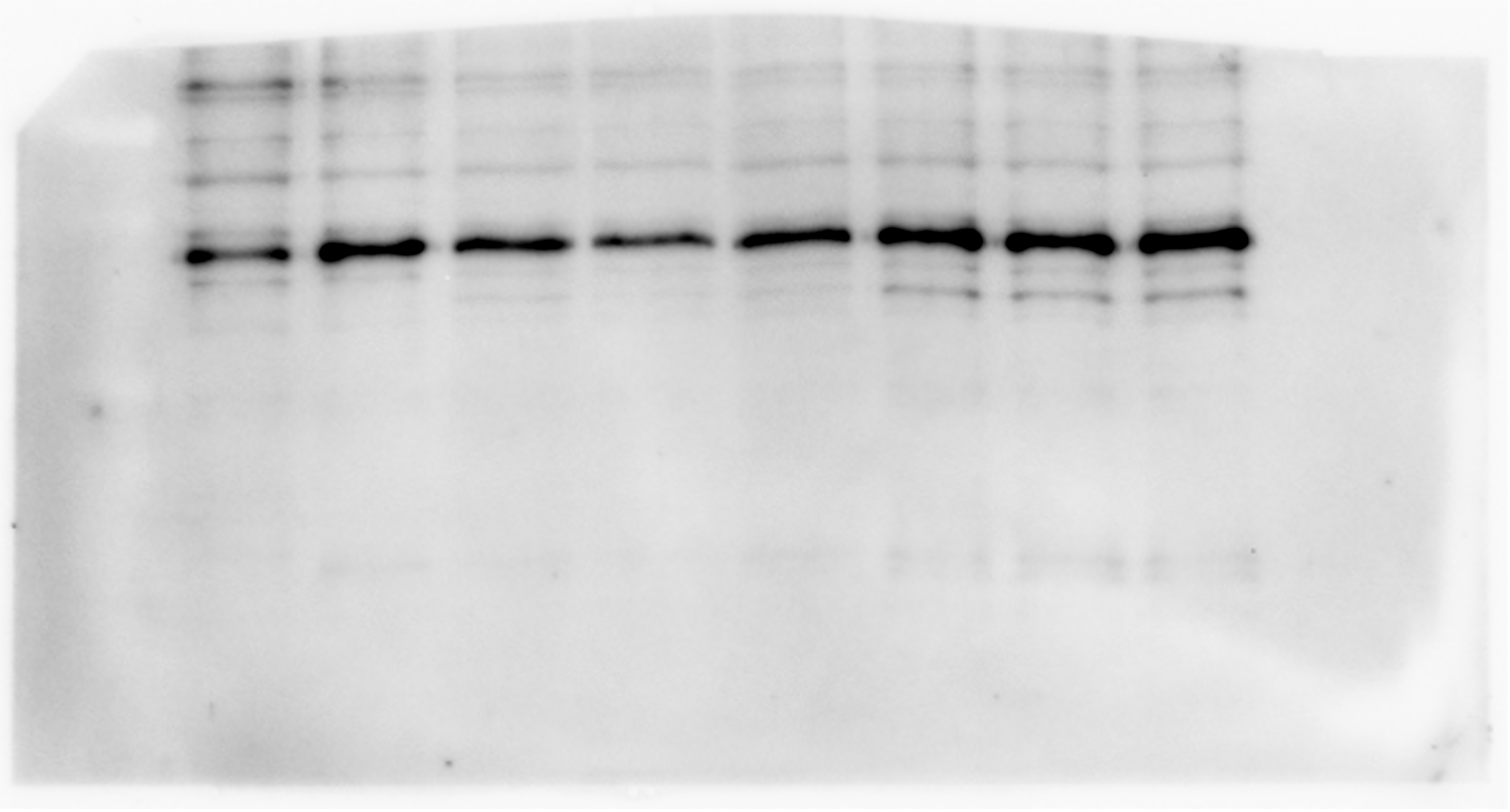

Supplement: Figure 6—source data 2. [file elife-107067-fig6-data2.zip › Fig6_Source_data2_raw/Fig6_SD2_raw4.png]

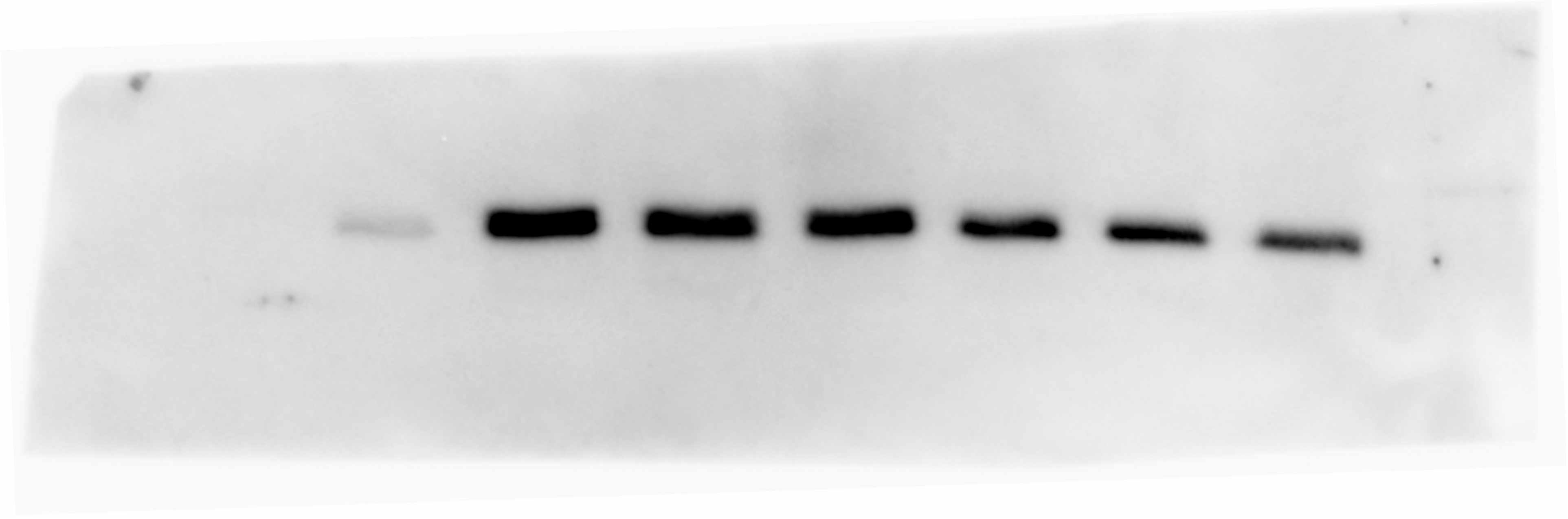

Supplement: Figure 6—source data 2. [file elife-107067-fig6-data2.zip › Fig6_Source_data2_raw/Fig6_SD2_raw3.png]

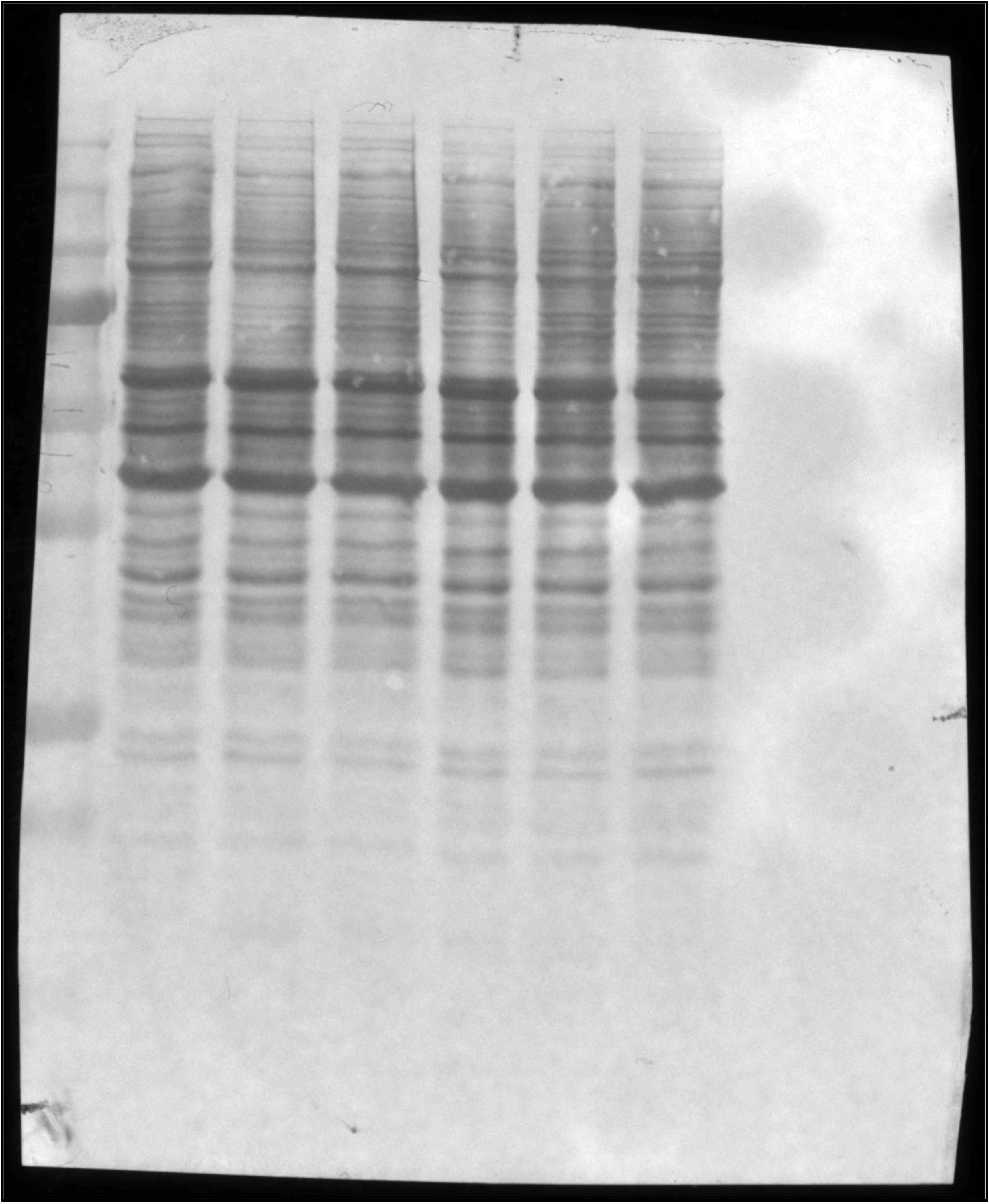

Supplement: Figure 6—source data 2. [file elife-107067-fig6-data2.zip › Fig6_Source_data2_raw/Fig6_SD2_raw2.png]

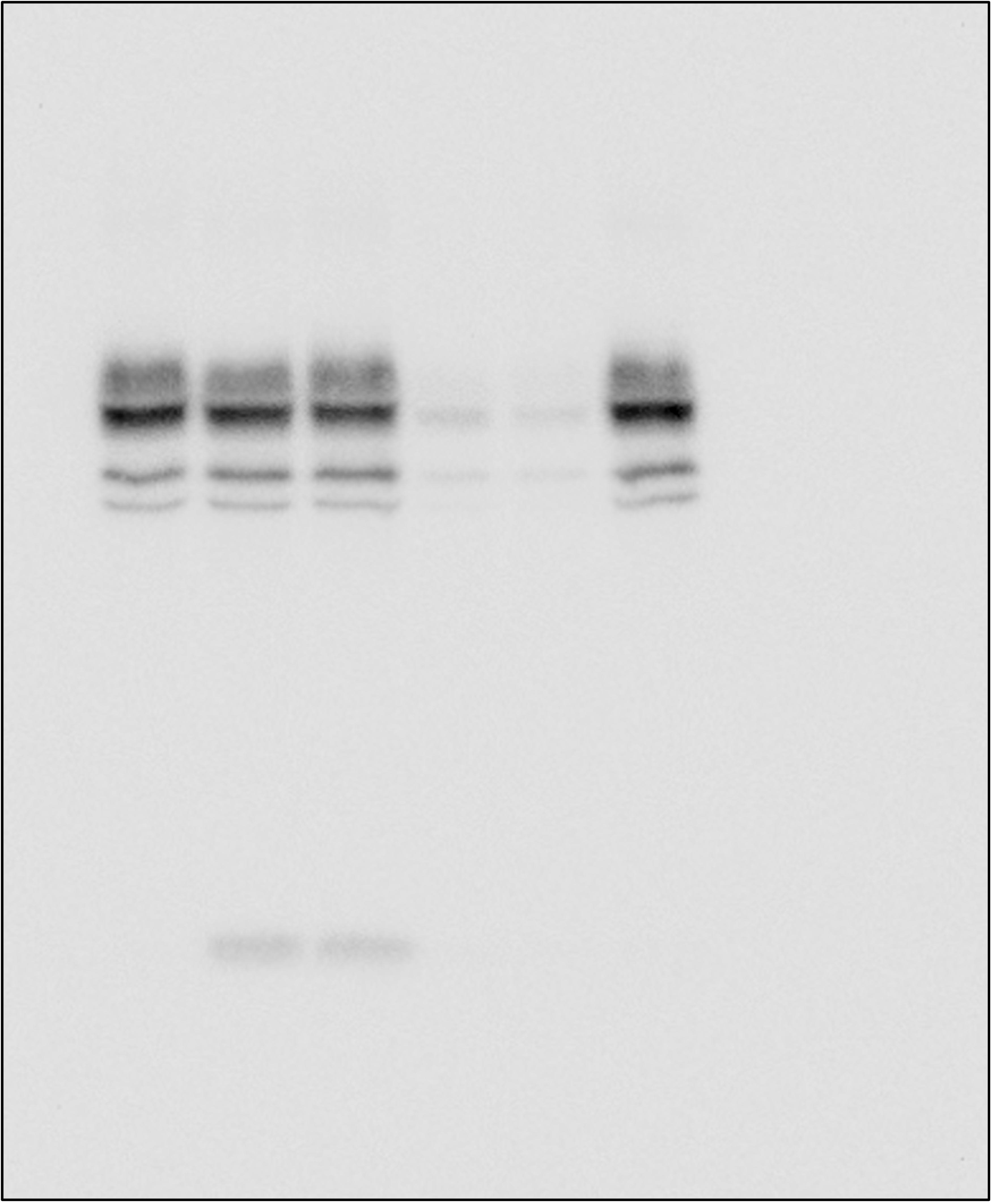

Supplement: Figure 6—source data 2. [file elife-107067-fig6-data2.zip › Fig6_Source_data2_raw/Fig6_SD2_raw1.png]

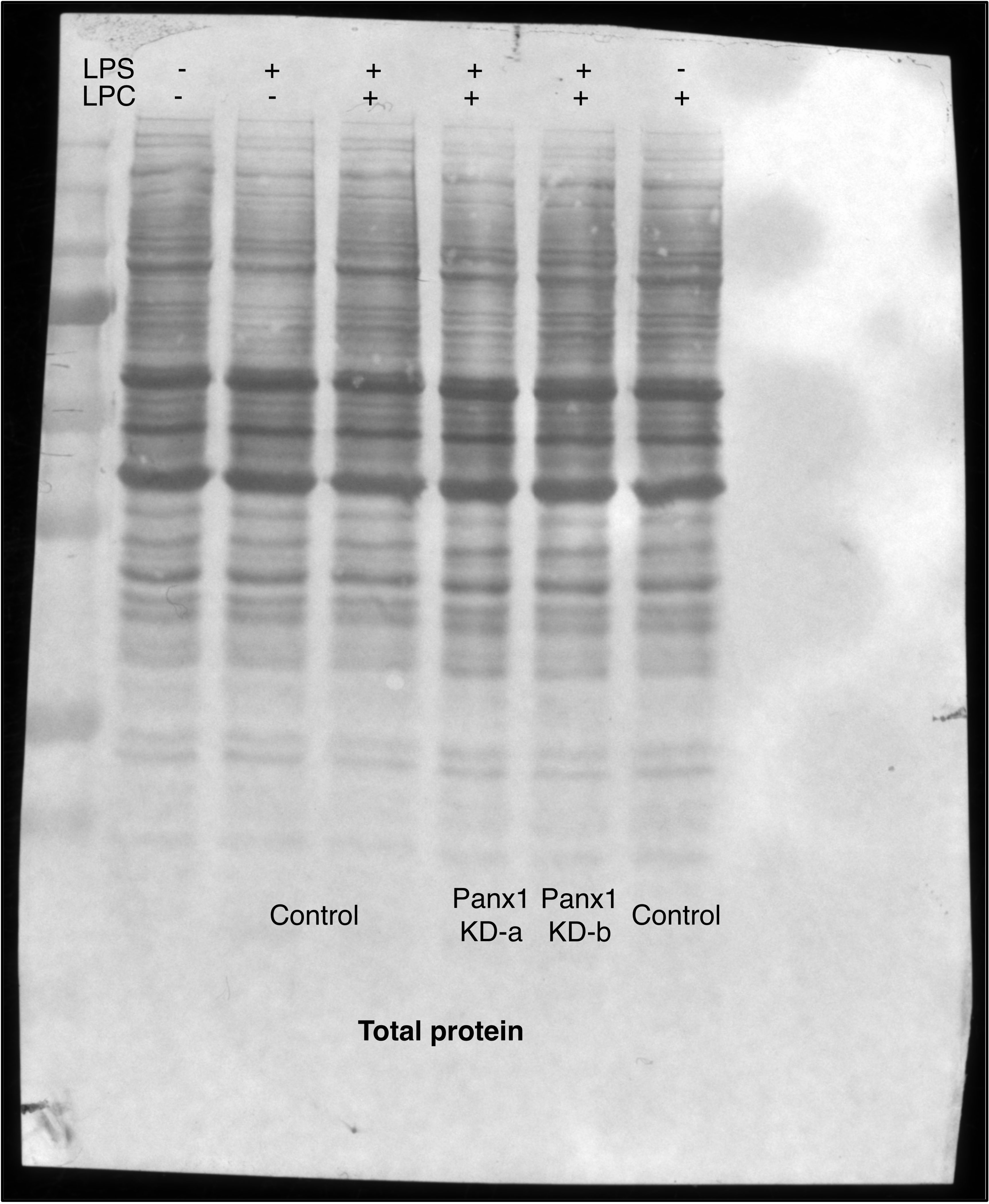

Supplement: Figure 6—source data 3. [file elife-107067-fig6-data3.zip › Fig6_Source_data3_annotated/Fig6_SD3_annotated2.png]

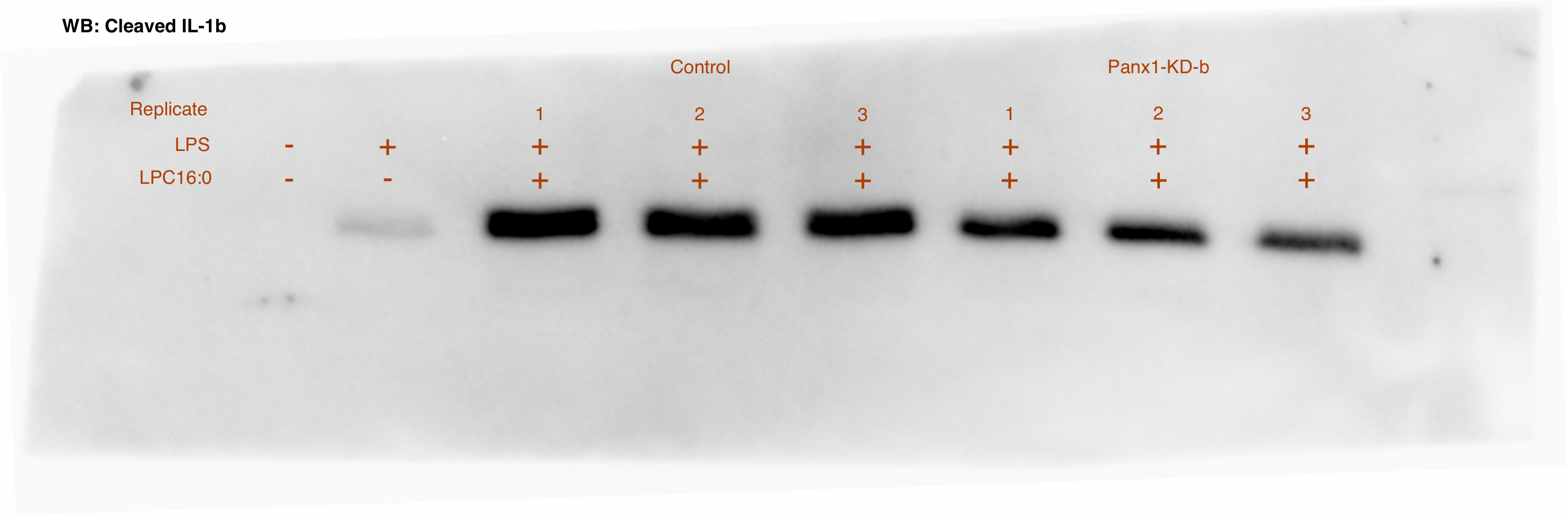

Supplement: Figure 6—source data 3. [file elife-107067-fig6-data3.zip › Fig6_Source_data3_annotated/Fig6_SD3_annotated3.png]

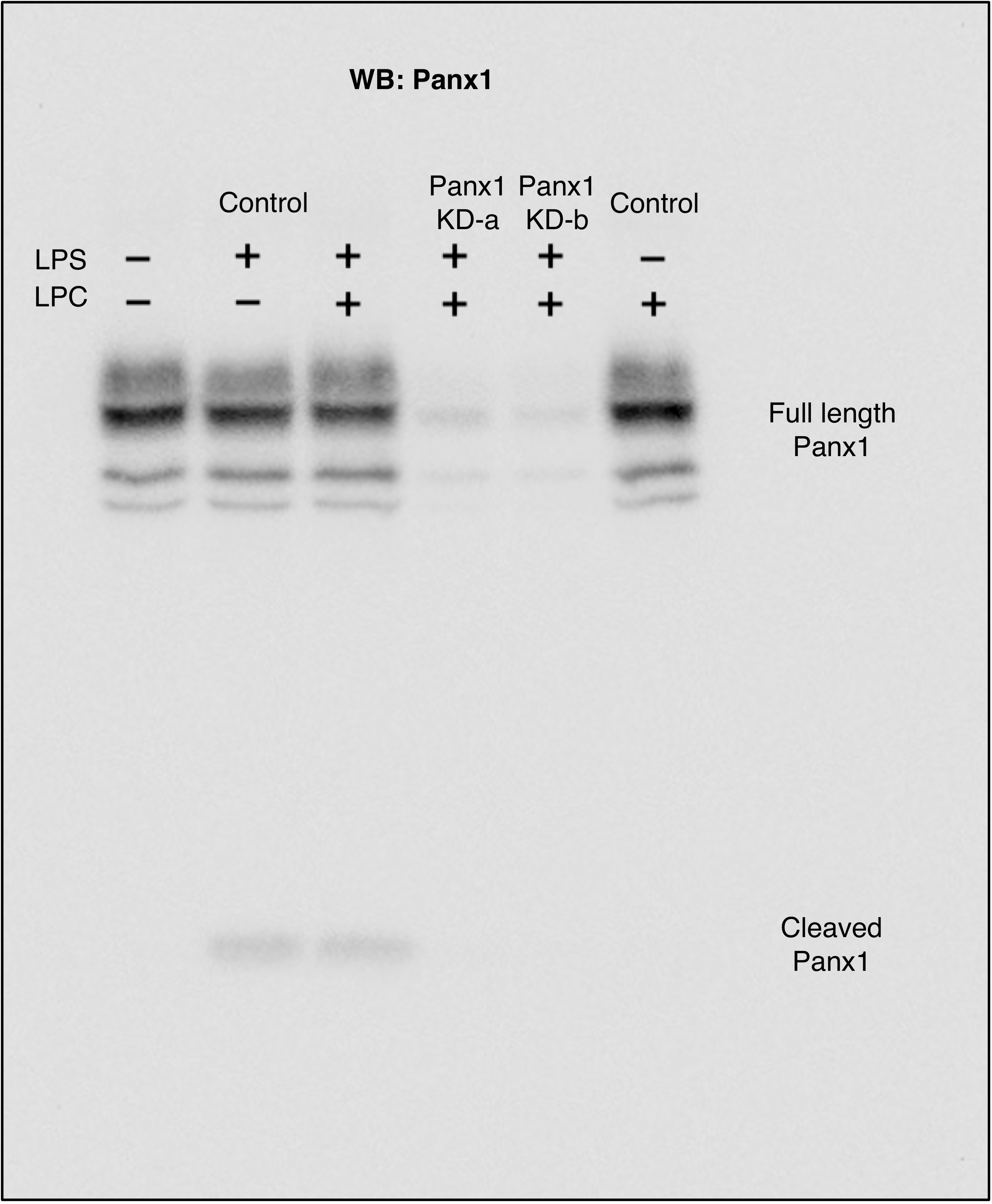

Supplement: Figure 6—source data 3. [file elife-107067-fig6-data3.zip › Fig6_Source_data3_annotated/Fig6_SD3_annotated1.png]

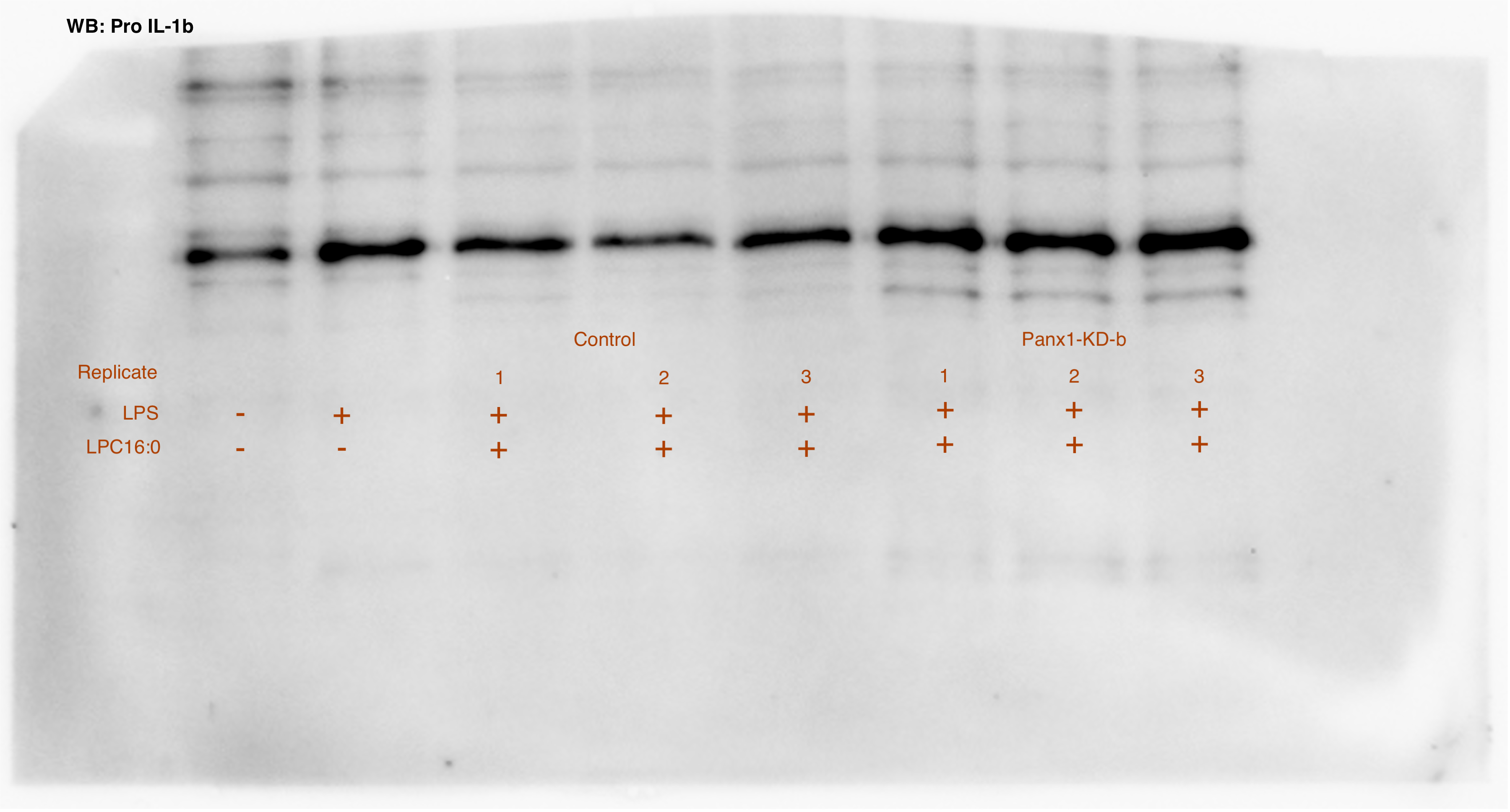

Supplement: Figure 6—source data 3. [file elife-107067-fig6-data3.zip › Fig6_Source_data3_annotated/Fig6_SD3_annotated4.png]

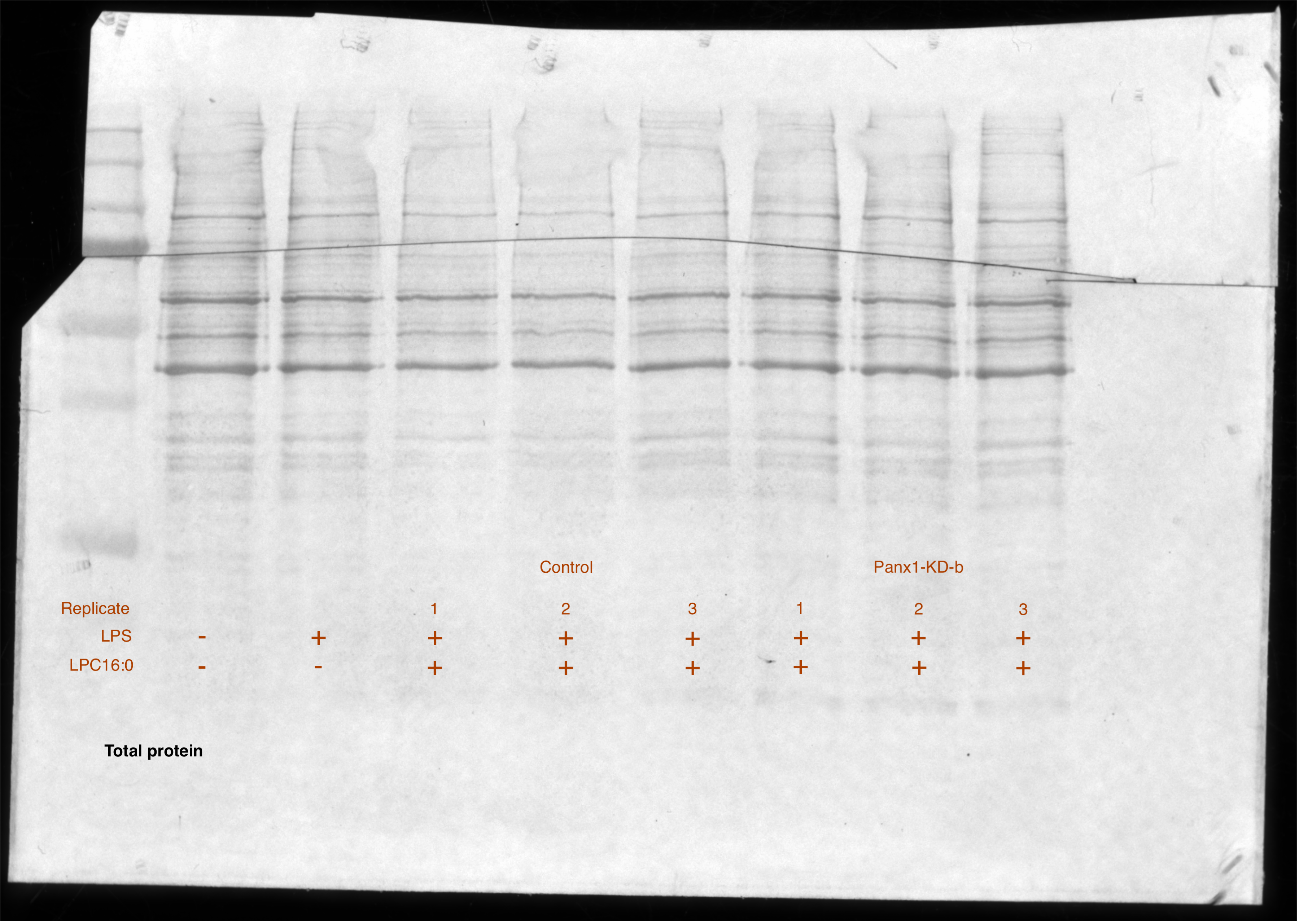

Supplement: Figure 6—source data 3. [file elife-107067-fig6-data3.zip › Fig6_Source_data3_annotated/Fig6_SD3_annotated5.png]

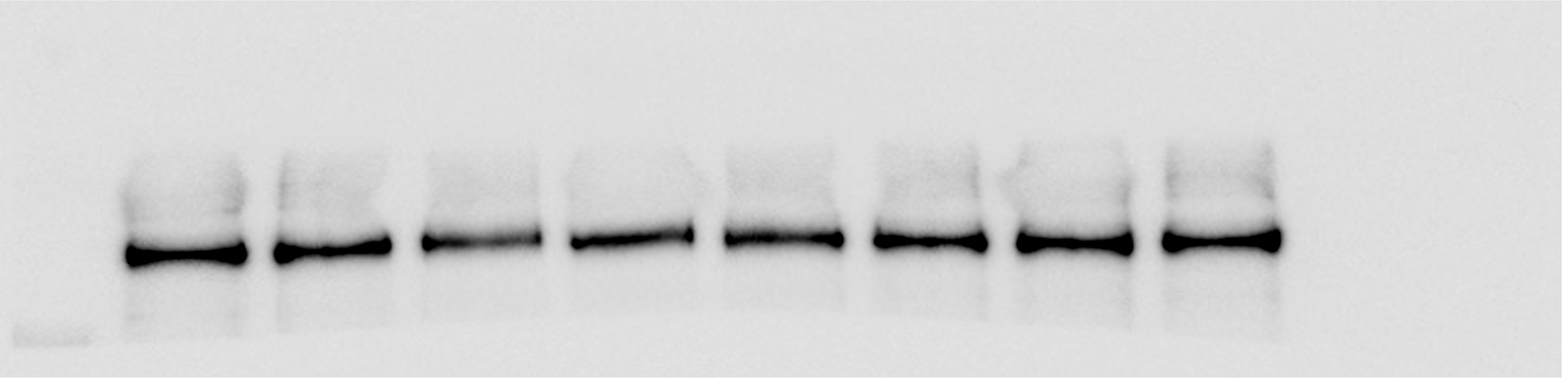

Supplement: Figure 6—figure supplement 1—source data 1. [file elife-107067-fig6-figsupp1-data1.zip › Fig6_Source_data4_raw/Fig6_SD4-raw1.png]

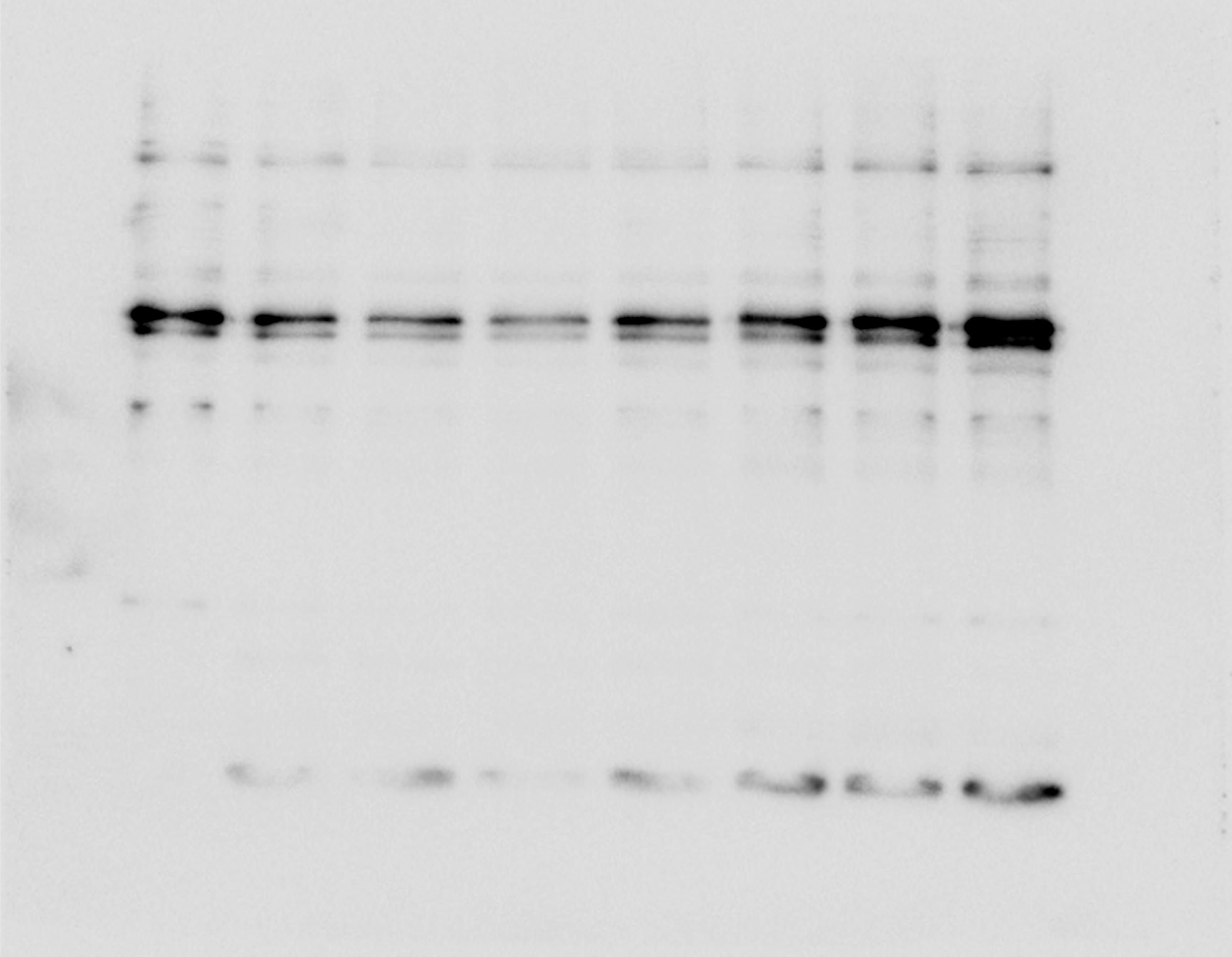

Supplement: Figure 6—figure supplement 1—source data 1. [file elife-107067-fig6-figsupp1-data1.zip › Fig6_Source_data4_raw/Fig6_SD4-raw3.png]

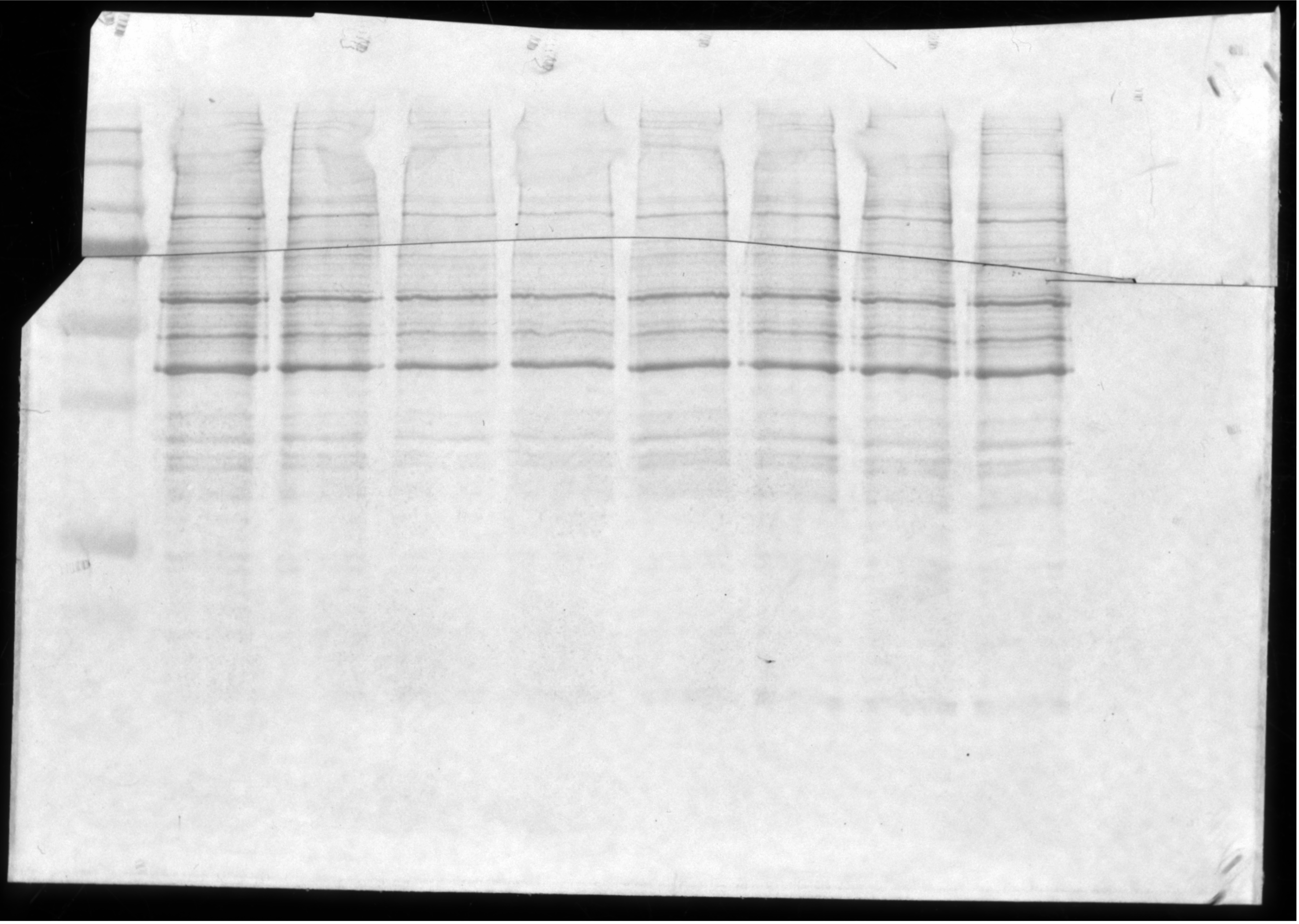

Supplement: Figure 6—figure supplement 1—source data 1. [file elife-107067-fig6-figsupp1-data1.zip › Fig6_Source_data4_raw/Fig6_SD4-raw2.png]

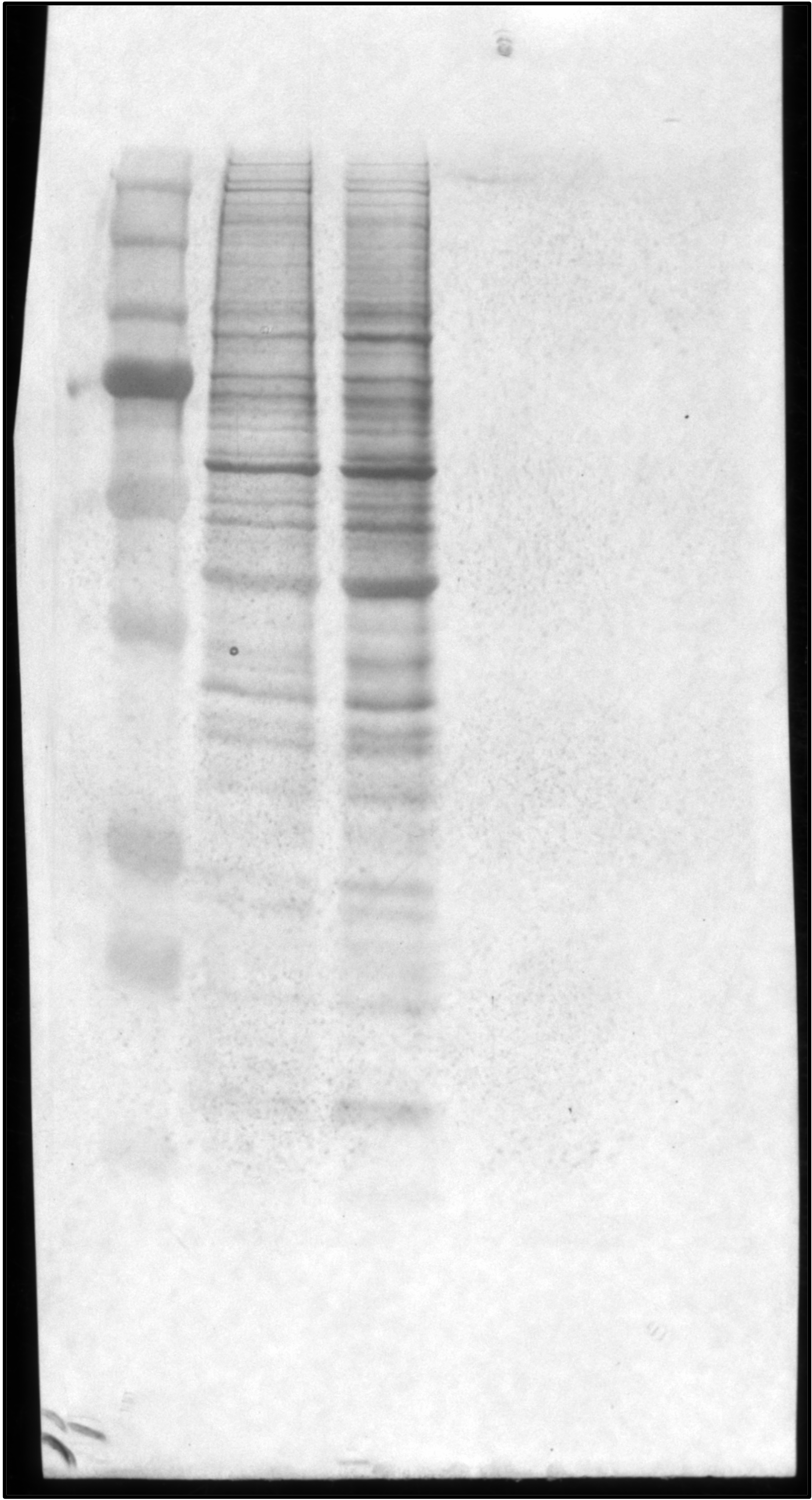

Supplement: Figure 6—figure supplement 1—source data 1. [file elife-107067-fig6-figsupp1-data1.zip › Fig6_Source_data4_raw/Fig6_SD4-raw6.png]

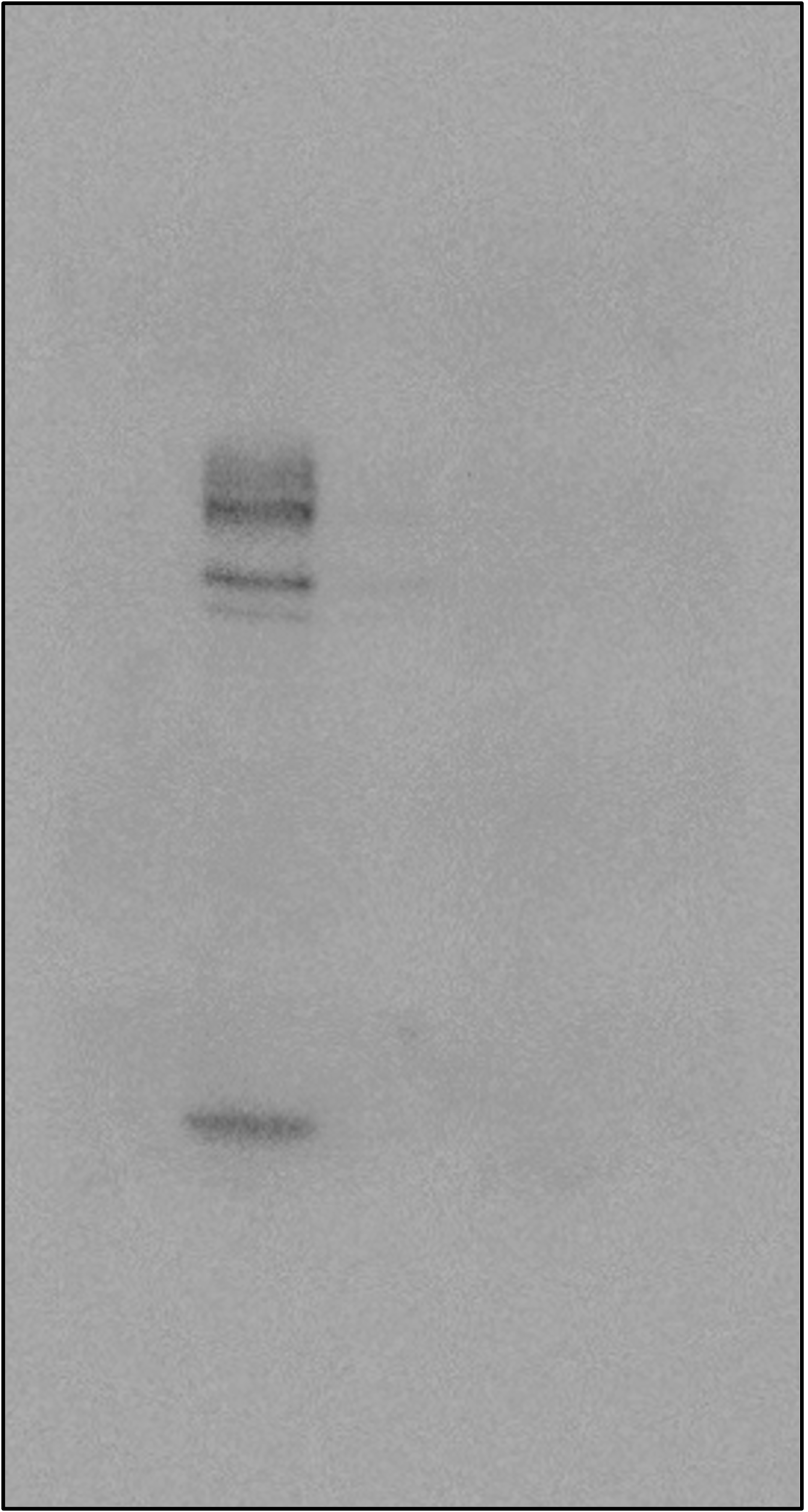

Supplement: Figure 6—figure supplement 1—source data 1. [file elife-107067-fig6-figsupp1-data1.zip › Fig6_Source_data4_raw/Fig6_SD4-raw5.png]

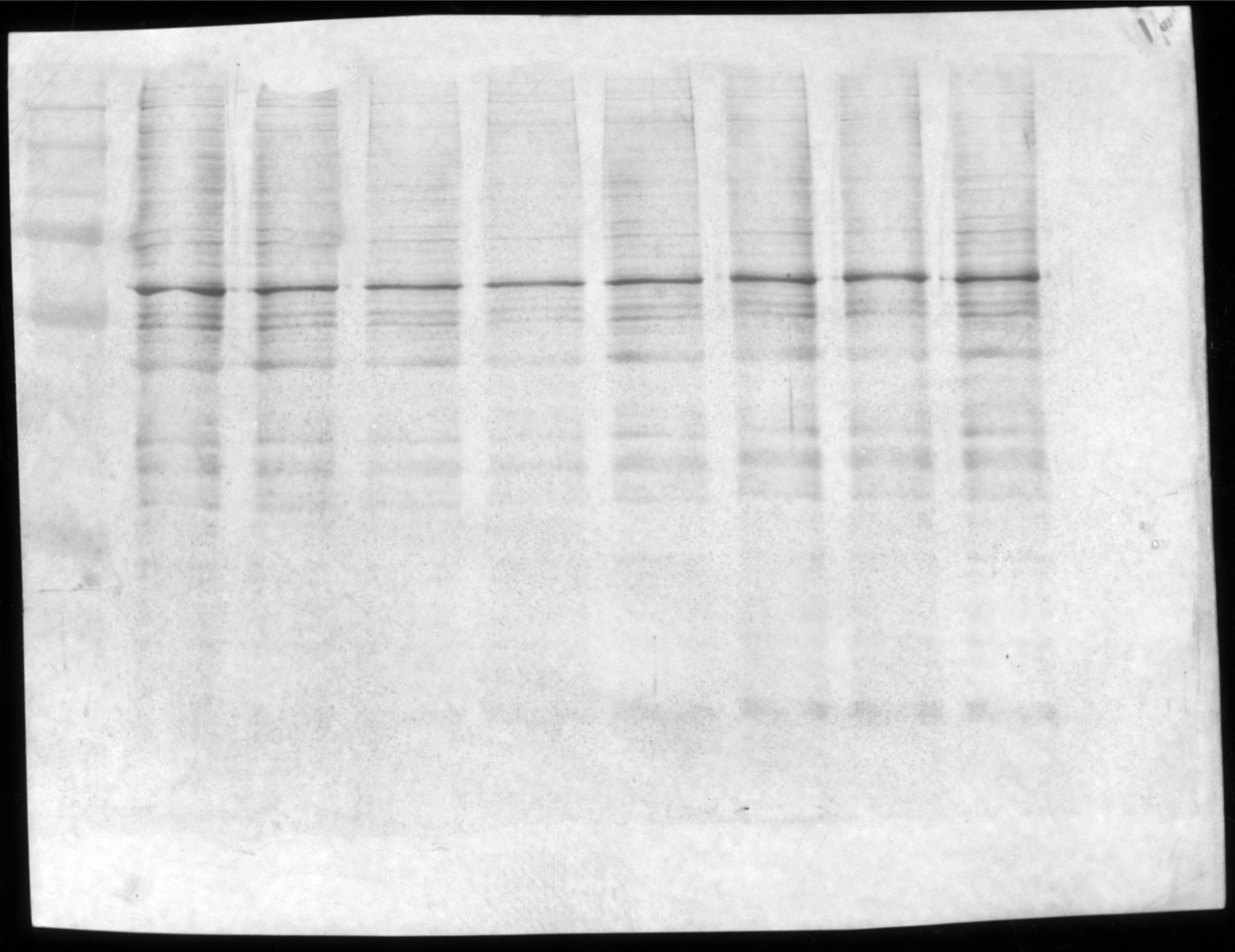

Supplement: Figure 6—figure supplement 1—source data 1. [file elife-107067-fig6-figsupp1-data1.zip › Fig6_Source_data4_raw/Fig6_SD4-raw4.png]

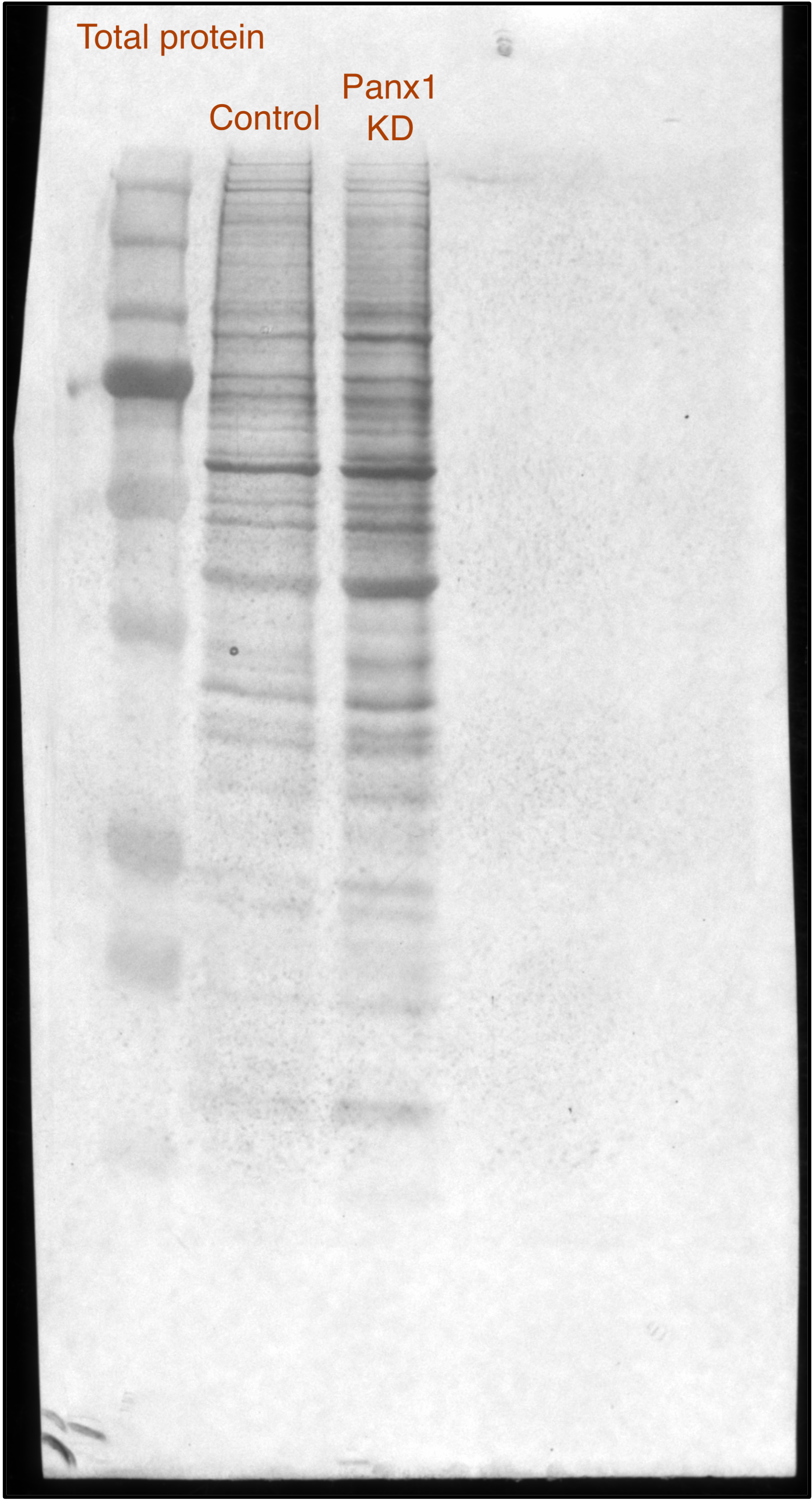

Supplement: Figure 6—figure supplement 1—source data 2. [file elife-107067-fig6-figsupp1-data2.zip › Fig6_Source_data5_annotated/Fig6_SD5-annotated6.png]

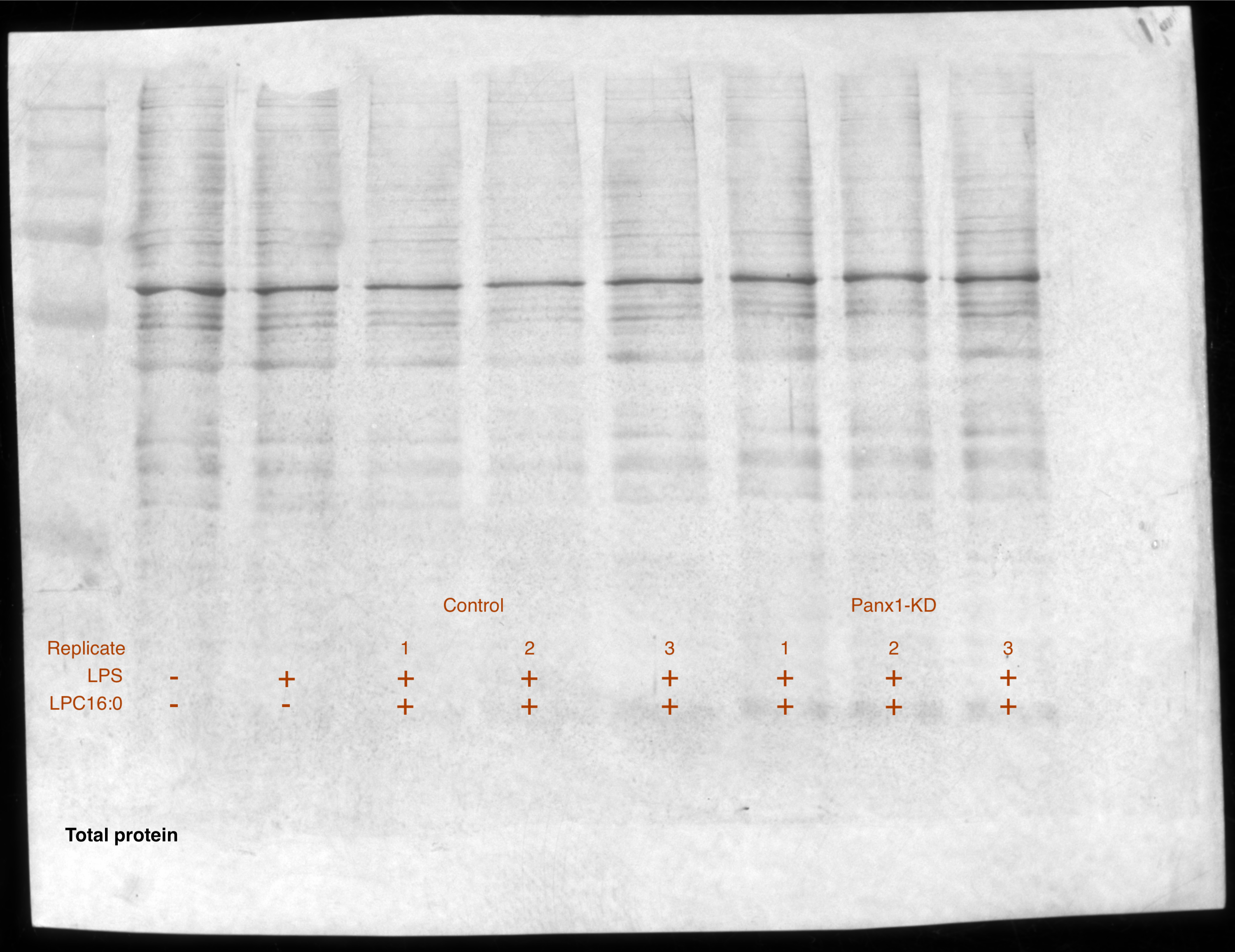

Supplement: Figure 6—figure supplement 1—source data 2. [file elife-107067-fig6-figsupp1-data2.zip › Fig6_Source_data5_annotated/Fig6_SD5-annotated4.png]

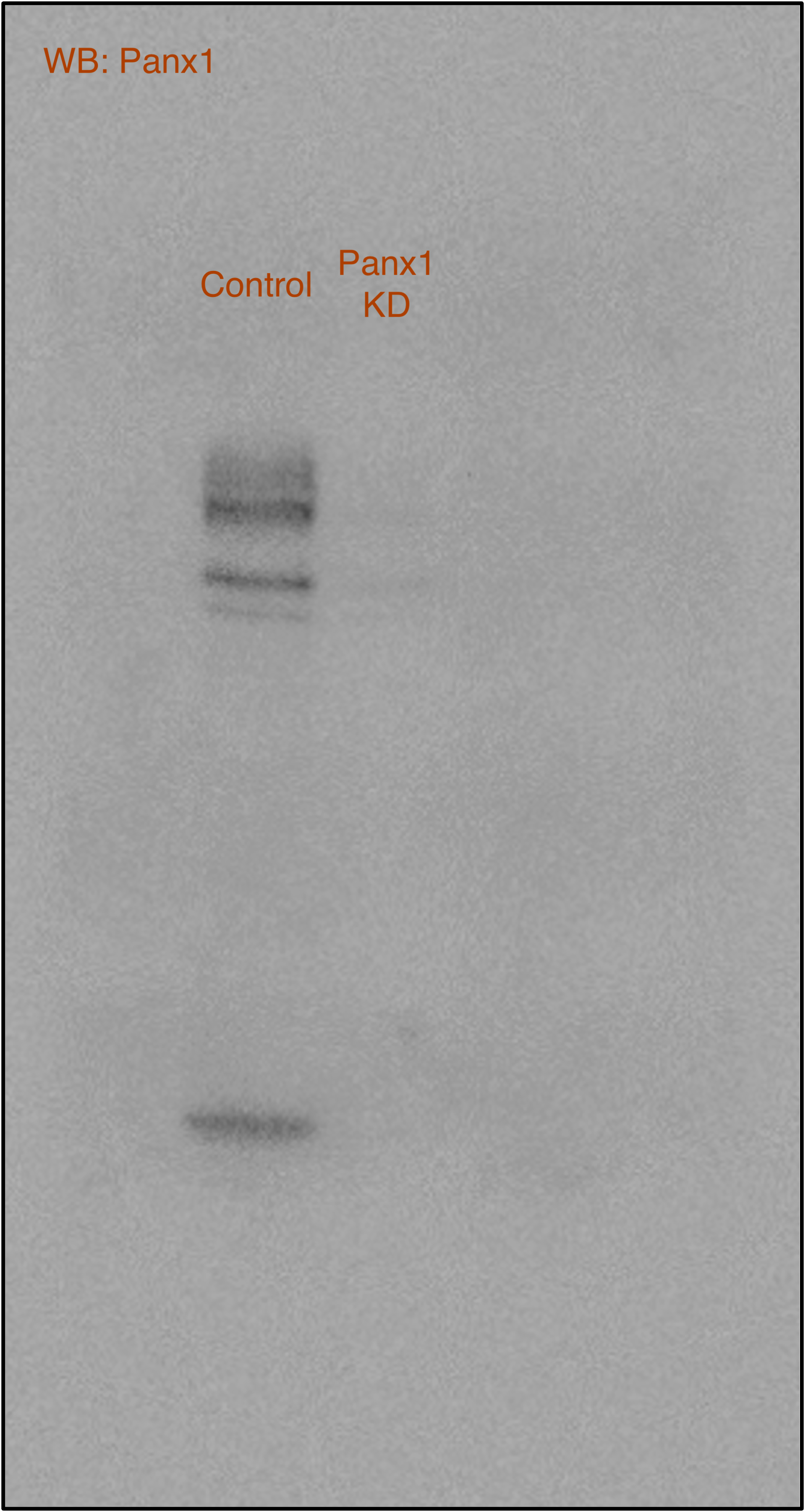

Supplement: Figure 6—figure supplement 1—source data 2. [file elife-107067-fig6-figsupp1-data2.zip › Fig6_Source_data5_annotated/Fig6_SD5-annotated5.png]

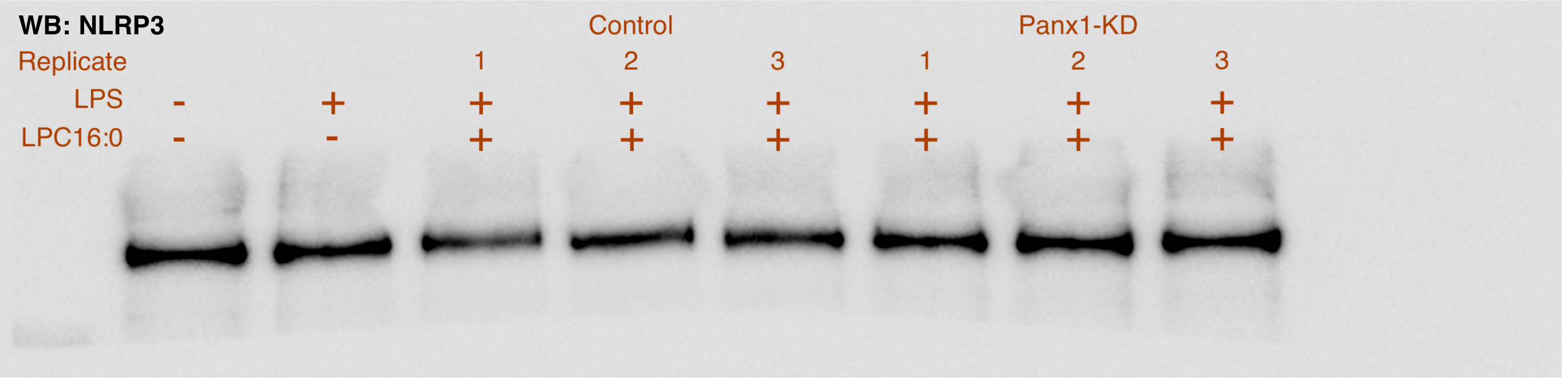

Supplement: Figure 6—figure supplement 1—source data 2. [file elife-107067-fig6-figsupp1-data2.zip › Fig6_Source_data5_annotated/Fig6_SD5-annotated1.png]

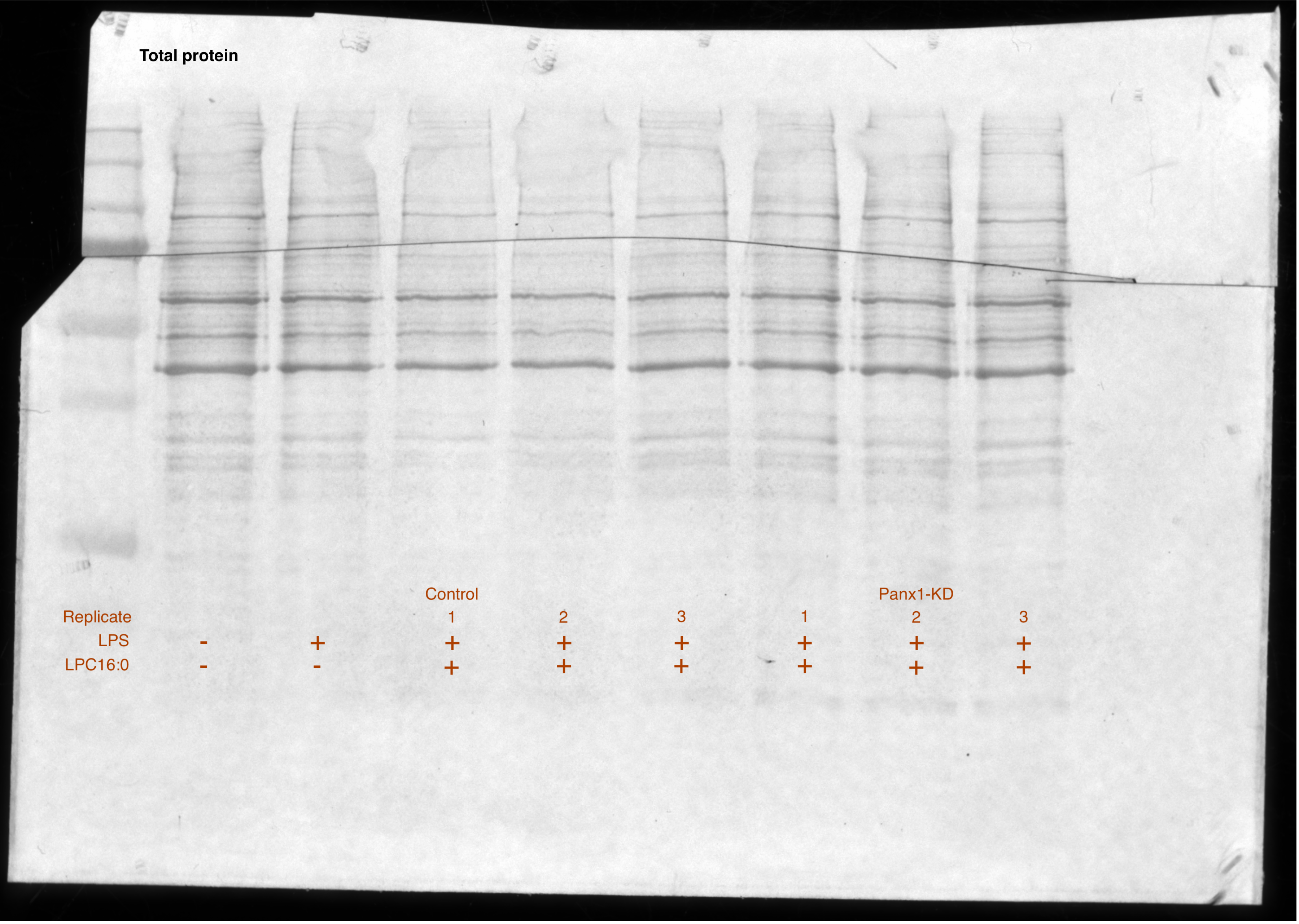

Supplement: Figure 6—figure supplement 1—source data 2. [file elife-107067-fig6-figsupp1-data2.zip › Fig6_Source_data5_annotated/Fig6_SD5-annotated2.png]

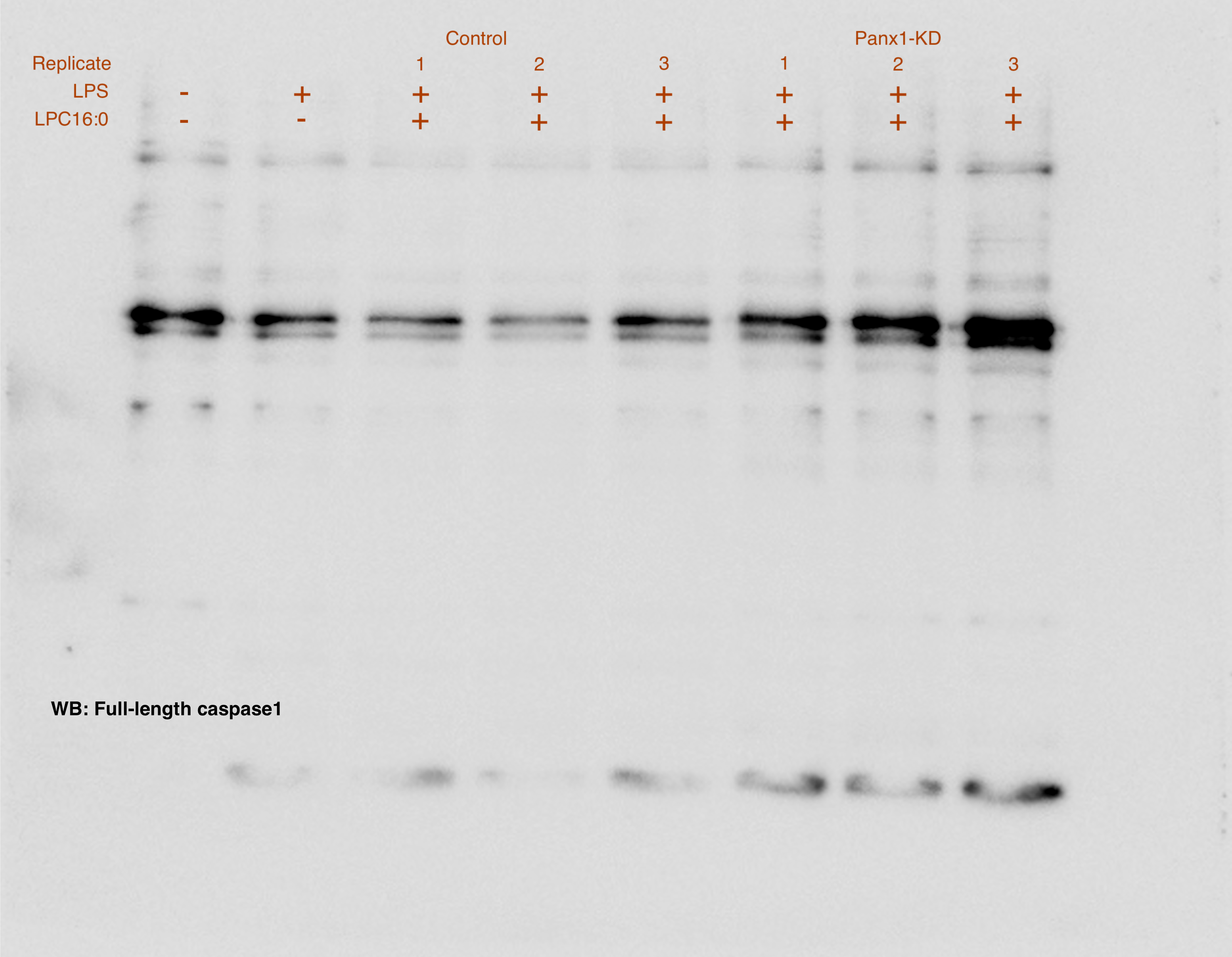

Supplement: Figure 6—figure supplement 1—source data 2. [file elife-107067-fig6-figsupp1-data2.zip › Fig6_Source_data5_annotated/Fig6_SD5-annotated3.png]
